# Neural Responses to Novel and Existing Words in Children with Autism Spectrum and Developmental Language Disorder

**DOI:** 10.5334/joc.204

**Published:** 2022-01-27

**Authors:** Victoria C. P. Knowland, Daniel H. Baker, M. Gareth Gaskell, Elaine van Rijn, Sarah A. Walker, Courtenay F. Norbury, Lisa-Marie Henderson

**Affiliations:** 1 Department of Psychology, University of York, York, YO10 5DD, UK; 2 Psychology and Language Sciences, University College London, UK

**Keywords:** ERP, N400, phonology, ASD, DLD

## Abstract

The formation of new phonological representations is key in establishing items in the mental lexicon. Phonological forms become stable with repetition, time and sleep. Atypicality in the establishment of new word forms is characteristic of children with developmental language disorder (DLD) and autism spectrum disorder (ASD), yet neural changes in response to novel word forms over time have not yet been directly compared in these groups.

This study measured habituation of event-related-potentials (ERPs) to novel and known words within and between two sessions spaced 24 hours apart in typically developing (TD) children, and their peers with DLD or ASD. We hypothesised that modulation of the auditory N400 amplitude would mark real-time changes in lexical processing with habituation evident within and across sessions in the TD group, while the DLD group would show attenuated habituation within sessions, and the ASD group attenuated habituation between sessions.

Twenty-one typically developing children, 19 children with ASD, and 16 children with DLD listened passively to known and novel words on two consecutive days, while ERPs were recorded using dry electrodes. Counter to our hypotheses, no habituation effect emerged within sessions. However, responses did habituate between sessions, with this effect being reduced in the DLD group, indicating less pre-activation of lexical representations in response to words encountered the previous day. No differences in change over time were observed between the TD and ASD groups. These data are in keeping with theories stressing the importance of sleep-related consolidation in word learning.

## Introduction

To learn a new word, the representation of a novel phonological form must become robust. While recognition is often possible after a single hearing, accurate retrieval benefits from repeated exposure (Roediger & Butler, 2011) and the integration of the new form with existing lexical knowledge. This latter process is known to be supported by sleep in both adults (Dumay & Gaskell, 2007) and children (Henderson et al., 2012; Landi et al., 2018). The process of word learning shows substantial variability across children, and is atypical in some groups with neurodevelopmental disorders, including developmental language disorder (DLD) and autism spectrum disorders (ASD). In this data report we present an experimental dataset designed to track change in the initial representation of novel phonological forms over 24 hours in children with DLD, ASD and their language-typical peers.

DLD is characterised by a difficulty in the establishment and use of age-typical language skills in both receptive and expressive modalities and at any level of language description. Weaknesses in vocabulary, syntax and phonological learning commonly co-occur in this population (see Bishop et al., 2016). Initial evidence suggests that early encounters with novel word forms result in atypical encoding in children with DLD (Bishop & Hsu, 2015). For example, this group shows well-established deficits in the ability to repeat novel pseudowords (non-word repetition; as first shown by Gathercole & Baddeley, 1990). Non-word repetition is thought to mimic early encounters with new words before semantic knowledge is established, with the quality of early representation within the phonological store being the foundation for subsequent building of a lexical item (Gathercole et al., 1997). Notably however, when tested on the same items an hour after first exposure, children with DLD show off-line maintenance of new word forms equivalent to typical controls (Bishop et al., 2012), suggesting initial encoding of phonological forms may be more difficult for these children than longer term maintenance of new phonology.

The process of encoding and consolidating new phonological forms can also be considered at the neural level. With repeated presentation of any stimulus, neurophysiological responses habituate. Such habituation is thought to result from increased processing efficiency (Grill-Spector et al., 2006) of items in short term memory storage, and is evident across multiple signatures of brain activity. Of particular relevance to the processing of linguistic stimuli is the N400, a negative-going event-related potential (ERP) component maximal over central sites 300–500 ms after stimulus onset. The N400 is understood to represent a response to meaningful or potentially meaningful stimuli presented in auditory or visual domains, reflecting lexical-semantic activation (Delogu et al., 2019; and see Kutas & Federmeier, 2011), and can evidence change in learning status before behavioural change is observed (McLaughlin et al., 2004). N400 amplitude is taken as an index of the difficulty of retrieving stored conceptual knowledge through the activation of possible lexical items from the point of word onset (Delogu et al., 2019). Stronger responses are seen when the retrieval of lexical information is more taxing on account of the word form being unknown, unexpected, or less familiar (Holcomb & Neville, 1990; Rugg, 1990). With repetition, a previously unknown item becomes more familiar and thereby elicits a smaller N400 response. Such habituation is observed with immediate repetition (Rugg, 1985), likely reflecting short-term storage for the recognition of recently encountered items, but is also observed over the longer term, reflecting changes in familiarity (McLaughlin et al., 2004) and ease of access.

Modulation of auditory N400 amplitude is sensitive to difficulties in the retrieval of lexical information in children with ASD (DiStefano et al., 2019; McCleery et al., 2010) and DLD (Kornilov et al., 2015), making it a useful tool with which to measure the real-time emergence of word learning in these groups (Nordt et al., 2016). Indeed, a similar measure has already been used to support the idea of reduced encoding of newly encountered phonological forms in children with DLD. The habituation of the N400 m (the magnetoencephalographic equivalent of the N400) has been shown to be absent in response to the second of two presentations of a novel word form in the left hemisphere of children with DLD relative to typically developing (TD) children (Helenius et al., 2014), suggesting the rapid decay of neural representations of novel word forms in this group.

Children with ASD show developmental difficulties in the social use of language, but also often have deficits in structural language and vocabulary (Marini et al., 2020; Williams et al., 2008). By contrast to children with DLD, verbally-able children on the autism spectrum show enhanced sensitivity to novel phonological forms immediately after exposure (Henderson et al., 2014) and are better at matching novel phonological forms to referents (Norbury et al., 2010) relative to typically developing vocabulary-matched peers. This population also shows non-word repetition abilities in-line with TD children (Williams et al., 2012). However, lexical representations are not thought to be typical in children with ASD, with behavioural evidence suggesting a loss of lexical (Henderson et al., 2014) and semantic (Fletcher et al., 2020; Norbury et al., 2010) knowledge about new words over time. Such difficulties in semantic memory consolidation in ASD have been linked to atypicalities in sleep parameters (Fletcher et al., 2020).

So while both DLD and ASD groups show deficits in vocabulary development, differences in early encoding of phonological form seems to be characteristic of DLD, while in ASD differences may emerge during the consolidation process. Comparing these groups therefore provides a natural experiment for testing hypotheses about the factors that are important early in the course of vocabulary acquisition, such as the role of sleep in supporting the establishment of new phonological forms. Neural changes in response to novel word forms over time have not yet been directly compared in these groups.

Here, we assess changes in neural response to new word forms in children who are developing typically compared to peers with DLD or ASD. We measure the maturation of the N400 response over the course of two sessions 24 hours apart in these three groups of children to examine whether responses to the same items habituate within each session, and also across sessions separated by a night of sleep.

Hypotheses

1. The typically developing group will show habituation of the N400 (less negative responses) with each presentation of a stimulus (Helenius et al., 2014; Minamoto et al., 2001).
2. The typically developing group will show greater habituation of the N400 on day two for novel words compared to known words (Davis & Gaskell, 2009).
3. The encoding of novel words within sessions will be less evident in children with DLD compared to typically developing peers, indexed by reduced habituation of the N400 in response to novel words (Helenius et al., 2014).
4. Children with ASD will show reduced consolidation of novel items between sessions, indexed by reduced habituation of the N400 between days compared to the typically developing and DLD groups (Fletcher et al., 2020; Henderson et al., 2014; Norbury et al., 2010).

## Method

### Participants

**Figure 1:**
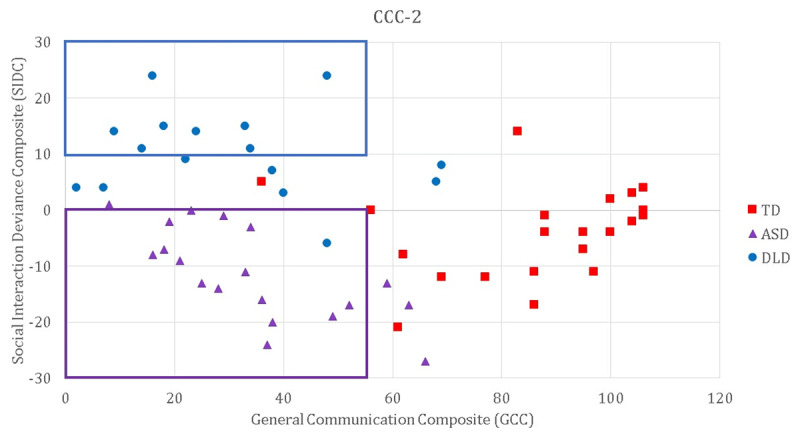
Children’s Communication Checklist (2^nd^ edition) scores. A GCC score below 55 in conjunction with a negative SIDC score (marked by the purple box) indicates social communication difficulties consistent with autism spectrum conditions; a GCC score below 55 in conjunction with an SIDC of 10 or more (marked by the blue box) is consistent with a structural language difficulty.

**Figure 2:**
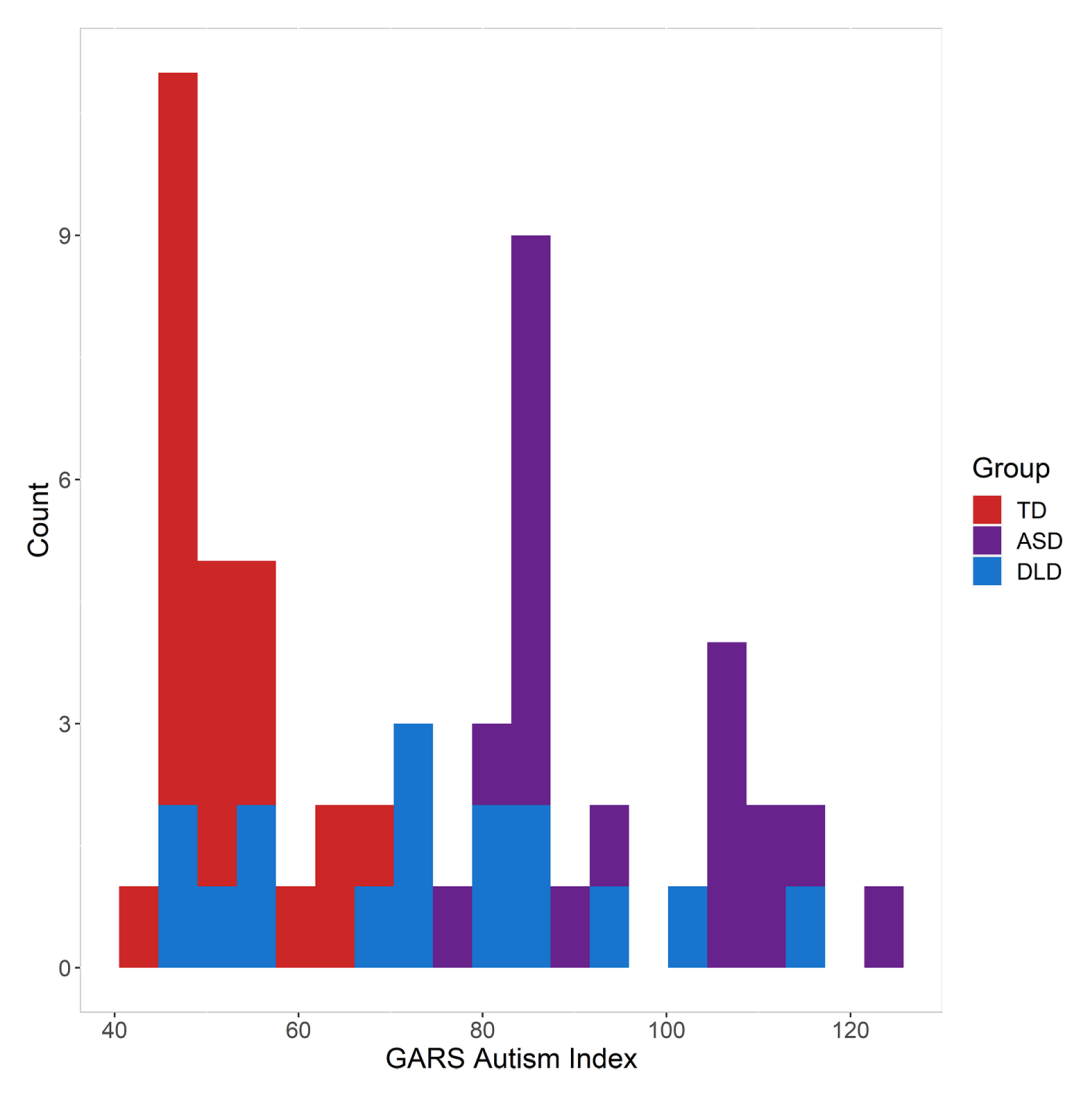
Autism Index scores on the GARS3 for the Typically Developing (TD; n = 21), Autism Spectrum Disorder (ASD; n = 19) and Developmental Language Disorder (DLD; n = 16) groups.

Fifty-eight participants were recruited from the UK through local schools and nationwide specialist schools as well as an existing database of children who had completed studies in the lab. Cognitive and developmental profiles were established through a set of standardised assessments and parent questionnaires (see ***Table 1***), including the Children’s Communication Checklist, 2^nd^ Edition (CCC-2; Bishop, 2003) and the Gilliam Autism Rating Scale, 3^rd^ Edition (GARS3, Gilliam, 2013), the distributions of which can be seen in ***Figures 1*** & ***2***, respectively. Twenty-one participants formed the typically developing (TD) group (age range: 8;7–12;8; see ***Table 1***). This group had no known developmental disorders and did not score below 10^th^ percentile on any standardised test in our cognitive battery. The DLD group included 16 children (age range = 7;11–12;3 years) with a diagnosis of DLD (or Specific Language Impairment) from a Speech and language Therapist. All participants in this group were receiving specialist intervention and/or scored below 10^th^ percentile on at least two standardised tests of language. Twenty-one children were recruited to the ASD group, though two did not complete testing on both days and were excluded, leaving 19 participants (age range = 8;10–13;0 years). Children were recruited to this group if they either had a diagnosis on the autism spectrum from an Educational Psychologist or multidisciplinary team (n = 16), or were awaiting diagnosis (n = 3), and scored within the ‘very likely’ range on the GARS3. Children in the ASD group had varying language ability. Children were not invited to participate if English was not their dominant language, if their parents or teachers reported a hearing deficit or if they were known to experience epileptic seizures.

**Table 1:** ***Descriptives***: n, mean (and SD) for Age (months), and Male:Female ratio; ***Standardised scores*** for each of the cognitive assessments administered: British Picture Vocabulary Scale, 3^rd^ Edition (BPVS-3); The Matrices, Word Definitions, and Backward Digit Recall subscales from the British Ability Scales, 3^rd^ Edition; Recalling Sentences subscale from the Clinical Evaluation of Language Fundamentals, 5^th^ Edition; Rapid Automatic Naming (RAN Digits) from the Comprehensive Test of Phonological Processing, 2nd Edition; ***Questionnaires*** mean (and SD) for Children’s Sleep Habits Questionnaire Sum score (CSHQ), Gilliam Autism Rating Scale Autism Index (GARS) and the GCC and SIDC scales from the CCC-2.


		TD	ASD	DLD	TD V ASD *T*	TD V DLD *T*

**Descriptives**	**n**	21	19	16	–	–

	**Age**	128.0 (15.2)	132.2 (15.8)	123.1 (17.3)	–	–

	**M:F**	15:6	15:4	7:9	–	–

**Standardised scores**	**BPVS–3**	110.0 (8.7)	102.6 (20.3)	76.2 (17.5)	1.82	7.08***

	**Recalling sentences**	111.0 (12.9)	97.1 (20.6)	70.6 (15.5)	2.51*	8.43***

	**Word definitions**	109.4 (13.2)	102.3 (19.5)	73.0 (19.4)	1.34	6.48***

	**B’ward digit span**	106.6 (18.0)	101.1 (14.7)	78.1 (13.6)	1.06	5.49***

	**Matrices**	111.5 (16.1)	102.7 (20.3)	77.5 (8.8)	1.51	8.20***

	**RAN digits**	107.4 (11.4)	90.8 (24.8)	79.7 (13.7)	2.67*	6.54***

**Questionnaires**	**CSHQ sum**	45.3 (15.7)	51.9 (6.6)	49.4 (7.0)	–1.77	–1.07

	**GARS Autism Index**	52.0 (6.6)	96.0 (14.2)	73.9 (19.5)	–12.32***	–4.29***

	**CCC_GCC**	86.0 (19.4)	34.4 (16.6)	30.6 (20.4)	9.05***	8.35***

	**CCC_SIDC**	–4.1 (8.1)	–11.6 (8.2)	10.1 (7.7)	2.88**	–5.45***


Two children were known to be taking melatonin to support sleep behaviour at the time of the study, one from the DLD group and one from the ASD group; children were not asked to refrain from their medication for the study.

### Protocol

Participants completed two sessions approximately 24 hours apart (mean = 23 hrs 27 mins, *SD* = 61 mins). The majority completed these sessions in their own home (14 TD; 17 ASD; 12 DLD), and a minority at the university (seven TD; two ASD; two DLD) or in school (two DLD). The study was granted ethical approval by the ethics committee for the Department of Psychology at the University of York.

A cognitive battery was administered to each participant (see ***Table 1***), comprising the following standardised assessments: British Picture Vocabulary Scale, 3^rd^ Edition (BPVS-3; Dunn et al., 2009); The Matrices, Word Definitions, and Backward Digit Recall subscales from the British Ability Scales, 3^rd^ Edition (BAS3; Elliott & Smith, 2011); Recalling Sentences subscale from the Clinical Evaluation of Language Fundamentals; Rapid Automatic Naming (RAN- Digits and Letters) from the Comprehensive Test of Phonological Processing, 2nd Edition (CTOPP2; Wagner et al., 2013). The parents of all children were asked to complete a series of questionnaires: The Children’s Sleep Habits Questionnaire (CSHQ, Owens et al., 2000); The CCC-2 and the GARS3.

### Stimuli and paradigms

#### ERP task

Based on the work of Helenius et al (2009; 2014), a mixed factorial design was adopted, with one between-subjects factor: Group (TD, DLD, ASD); and three within-subjects factors: Lexicality (Words, Pseudowords), Presentation (1, 2, 3), and Day 1, Day 2. Auditory stimuli for the ERP task were 50 real words and 50 pseudowords. For the Word stimuli, 50 concrete nouns were selected (see Table SM1), each 3-or-4 syllables in length with an age-of-acquisition of less than 8; 0 years (Kuperman et al., 2012).

Pseudowords were created from the Word stimuli (e.g., ALIBODO derived from ALLIGATOR) to minimise acoustic and phonotactic differences between conditions, while maintaining the lexical-semantic distinction. Pseudowords were onset-matched to their Word counterparts but diverged at the uniqueness point (UP, the point in a spoken word at which it becomes uniquely identifiable) (see Table SM2). UP varied between 127 ms and 527 ms after stimulus onset (mean = 313.7 ms, *SD* = 92.8); 40 Words and 39 Pseudowords had uniqueness points before 400 ms (on average 120 ms before).

**Figure 3:**
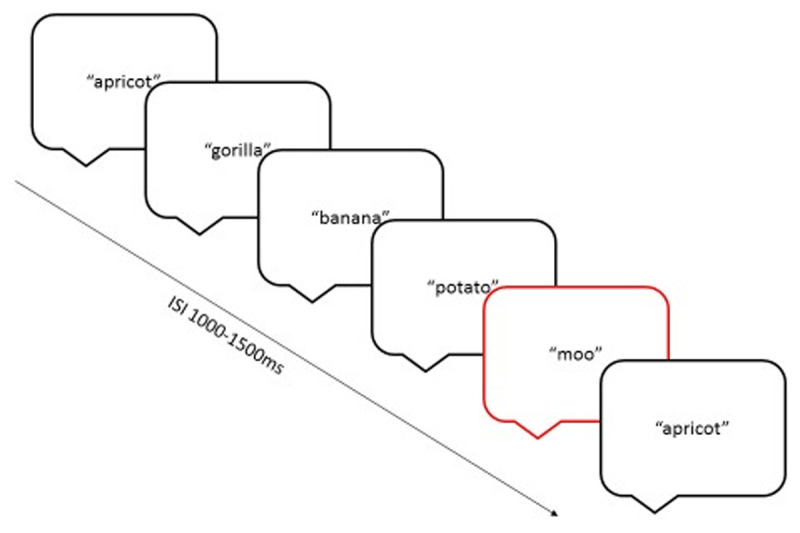
Example string of items. The item highlighted in red is a catch trial.

Each stimulus was presented three times, plus 32 catch trials, resulting in a total of 332 trials on each day (a maximum of 50 trials per cell). The three presentations of each item within a block were not sequential, but occurred with 5–10 intervening items (see ***Figure 3***). Inter-stimulus-interval was randomly jittered between 2,200–3,200 ms, and stimulus length varied between 626 and 1,289 ms (mean = 948 ms). The experiment was split into four blocks, with each block lasting approximately four minutes. Stimuli were delivered via over-the-ear headphones at a comfortable listening level, around 60 dB.

The order of the blocks was randomised across sessions but the order of stimuli within each block was kept consistent. Participants were not asked to respond to the stimuli as requiring a response from children may have placed different demands on each of the groups. However, given the passive nature of the task, to maintain and monitor children’s attention, they were required to listen for spoken animal noises (‘woof’, ‘moo’, ‘quack’ and ‘oink’), and to press a button when they heard one of these noises. Each animal noise was presented twice in each block. Children were not encouraged to press as fast as they could to avoid excessive motoric preparedness responses.

#### ERP recording

ERPs were recorded using a portable g.USBamp amplifier, with a sampling frequency of 1,200 Hz, with g.SAHARAsys dry active electrodes. Eight channels were used, four along the midline at FPz, Fz, Cz, and Pz, plus two lateral pairs at T7, T8 and C3, C4, anchored in an appropriately sized cap for each child. The reference electrode was placed on the left earlobe and the ground on the forehead. The experimental paradigm and electroencephalographic (EEG) recording were programmed in MATLAB (The MathWorks, 2014). EEG recording for each trial was triggered each time a trial was initiated, with a 687 ms period before the stimulus began; after which responses were recorded for 2,000 ms. A 200 ms baseline was used.

## Results

### Data processing

Artefact rejection was performed by excluding any trial where peak amplitude exceeded ± 100 μV relative to the 200 ms pre-stimulus baseline period. Data points were then rejected if they fell outside ±2 *SD* of the grand mean. The number of missing trials for remaining participants varied across groups. Of a possible 600 trials in total for each remaining participant, 67.9% of trials were maintained for the TD group, 72.6% for the ASD group and only 50.1% for the DLD group. It is likely that much of the loss in the DLD group can be attributed to movement artefacts with loss of attention, as indicated by the catch trial data. For the TD group, 2.10% of catch trials were lapses, for the ASD group 4.69%, and for the DLD group this rose to 13.57%, resulting in a significant group difference in lapses (χ^2^ = 78.026, *df* = 2, *p* < 0.001).

### Mixed effects model

**Figure 4:**
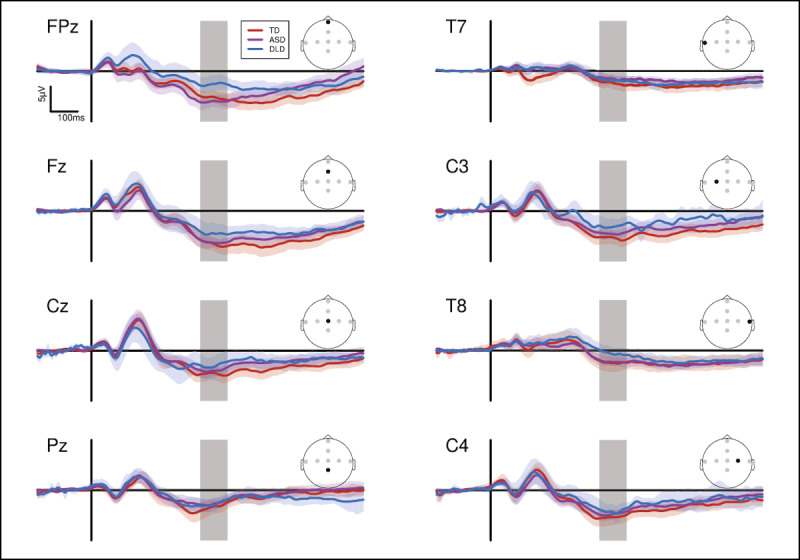
Grand average activity at each electrode for each group. Grey rectangles indicate 400–500 ms post stimulus onset. Shaded regions surrounding the curves indicate bootstrapped 95% confidence intervals.

Data were analysed in R (R Core Team, 2017) using a multilevel modelling approach (Volpert-Esmond et al., 2018). Models were built with ‘lme4’ (Bates et al., 2015) with plots made using ‘ggplot2’ (Wickham, 2016). The dependent variable was determined after visual inspection (by VK, LH, GG, DB) of grand mean averages for each electrode to establish the topography and timing of the N400 response (see Junge et al., 2012; 2021 for similar approaches to the establishment of temporal windows for analysis in ERP studies with developmental populations). Agreement was reached over the temporal window of the local minima around the vertex between 200–600 ms post stimulus onset (Kutas & Federmeier, 2011). Two linear mixed effects models were subsequently built with average amplitude over 400–500 ms post stimulus onset at four electrodes (Fz, Cz, C3 & C4) as the dependent variable (***Figure 4***). The temporal window used here is in line with previous work on the auditory N400 with children (Holcomb et al, 1992).

In order to address Hypotheses 1 & 2, the following factors were entered as fixed effects: Day (simple coding: Day 1 (–0.5), Day 2 (0.5)), Lexicality (simple coding: Words (–0.5), Pseudowords (0.5)), Presentation (forward difference coding: 1vs2 (1 (2/3), 2(–1/3), 3(–1/3), 2v3 (1/3, 1/3, –2/3), and Electrode (deviation coding with levels Cz, Fz, C3, C4: (–1,0,0,1), (–1,0,1,0), (–1,1,0,0)), the model included an interaction between Day and Lexicality and only considered data from the TD group. In order to address Hypotheses 3 & 4 data from all groups was considered. In addition to the fixed effects described above, Group (simple coding: TDvASD (TD(–1/3), ASD(2/3), DLD(–1/3), TDvDLD (TD(–1/3), ASD(–1/3), DLD(2/3)), was included along with the following interactions: Presentation:Group:Lexicality (Hypothesis 3); Group:Day:Lexicality (Hypothesis 4). Three-way interactions were excluded where the model experienced rank deficiency and were replaced with simpler two-way interactions, leaving the final models as described in ***Tables 2*** & ***3***. Significant interactions were explored post-hoc using the ‘emmeans’ package (Length, 2019), with Tukey correction for multiple comparisons. Subject was added as a random effect to both models, along with UP, as although auditory N400 research is typically based on time since stimulus onset, the UP defines point of lexical access (e.g., Gagnepain et al., 2012). Visual inspection of Q-Q plots confirmed no substantial deviation from heterogeneity of variance of the residuals.

**Table 2:** Fixed and random effects for model at 400–500 ms post stimulus onset. Model formed from 35,830 observations: 21 participants across 2 sessions. Significance levels: *** at p < 0.001, ** p < 0.001, * p < 0.05.


*FIXED EFFECTS*									*RANDOM EFFECTS*	
	
	*B*		95% CI			*T*	*P*		*SUBJECT*	*UP*
			
			*LOWER*	*UPPER*					SD	SD

(Intercept)	–6.37		–7.45	–5.30		–11.614	<0.001***		2.31	1.64

Day1v2	2.08		1.59	2.57		8.290	<0.001***			

Electrode Cz v Fz	0.15		–0.26	0.57		0.726	0.468			

Electrode Cz v C3	0.07		–0.34	0.48		0.341	0.733			

Electrode Cz v C4	–0.84		–1.26	–0.41		–3.844	<0.001***			

Presentation 1v2	0.06		–0.53	0.66		0.208	0.835			

Presentation 2v3	0.59		0.00	1.18		1.952	0.051			

Lexicality	–0.25		–1.01	0.52		–0.635	0.527			

Day:Lexicality	0.89		–0.08	1.86		1.799	0.072			


**Table 3:** Fixed and random effects for model at 400–500 ms post stimulus onset. Model formed from 90,805 observations: 56 participants across 2 sessions. Significance levels: *** at p < 0.001, ** p < 0.001, * p < 0.05.


*FIXED EFFECTS*									*RANDOM EFFECTS*	
	
	*B*		95% CI			*T*	*P*		*SUBJECT*	*UP*
			
			*LOWER*	*UPPER*					SD	SD

(Intercept)	–5.29		–5.88	–4.70		–17.586	<0.001***		2.01	0.97

Day1v2	1.83		1.52	2.15		11.288	<0.001***			

Group TDvASD	1.23		–0.06	2.53		1.863	0.068			

Group TDvDLD	1.95		0.58	3.31		2.790	0.007**			

Electrode Cz v Fz	0.05		–0.21	0.31		0.376	0.707			

Electrode Cz v C3	0.15		–0.12	0.41		1.091	0.275			

Electrode Cz v C4	–0.87		–1.13	–0.60		–6.401	<0.001***			

Presentation 1v2	0.07		–0.31	0.46		0.379	0.705			

Presentation 2v3	0.17		–0.21	0.55		0.891	0.373			

Lexicality	–0.64		–1.12	–0.17		–2.671	0.008**			

Day:Group(TDvASD)	0.39		–0.31	1.09		1.097	0.273			

Day:Group(TDvDLD)	–1.14		–1.95	–0.33		–2.742	0.006**			

Group(TDvASD):Pres. (1v2)	0.09		–0.77	0.94		0.201	0.841			

Group(TDvDLD):Pres. (1v2)	–0.01		–0.99	0.97		–0.014	0.989			

Group(TDvASD):Pres. (2v3)	–0.74		–1.59	0.11		–1.714	0.086			

Group(TDvDLD):Pres. (2v3)	–0.56		–1.52	0.41		–1.129	0.259			

Group(TDvASD):Lexicality	–1.28		–1.97	–0.58		–3.599	<0.001***			

Group(TDvDLD):Lexicality	–0.28		–1.08	0.51		–0.697	0.486			


#### Model 1

For the TD group data, a main effect of Day emerged, with response amplitude lower on Day 2 (Day 1 mean = –7.43 µV, *SE* = 0.17, Day 2 mean = –5.30 µV, *SE* = 0.18). A main effect of Electrode indicated a significant contrast between C4 and Cz (Cz mean = –5.82 µV, *SE* = 0.26; Fz mean = –7.29 µV, *SE* = 0.26; C3 mean = –6.33 µV, *SE* = 0.24; C4 mean = –6.23 µV, *SE* = 0.24).

Hypothesis 1 stated that *The typically developing group will show habituation of the N400 (less negative responses) with each presentation of stimuli*. As no main effect of Presentation emerged, the first hypothesis was not supported. Hypothesis 2 stated that *The typically developing group will show greater habituation of the N400 on day two for novel words compared to known words* such that an interaction was expected between Day and Lexicality. This interaction did not emerge, such that Hypothesis 2 was also not supported.

#### Model 2

As shown in ***Table 3***, a main effect of Group emerged, where the DLD group showed lower amplitude activity than the TD group (TD mean = –6.41 µV, *SE* = 0.12, DLD mean = –4.37 µV, *SE* = 0.17). Numerically, the ASD group showed lower amplitude activity than the TD group, but this was not significant (ASD mean = –5.07 µV, *SE* = 0.13). A main effect of Day was evident, with response amplitude lower on Day 2 (Day 1 mean = –6.43 µV, *SE* = 0.11, Day 2 mean = –4.40 µV, *SE* = 0.11). A main effect of Lexicality was seen, with Pseudowords evoking a higher amplitude (more negative) response (Words mean –5.22 µV, *SE* = 0.11, Pseudowords mean –5.65 µV, *SE* = 0.11). Finally, a main effect of Electrode indicated a significant contrast between C4 and Cz (Cz mean = –4.75 µV, *SE* = 0.16; Fz mean = –6.29 µV, *SE* = 0.16; C3 mean = –5.25 µV, *SE* = 0.15; C4 mean = –5.41 µV, *SE* = 0.15).

**Figure 5:**
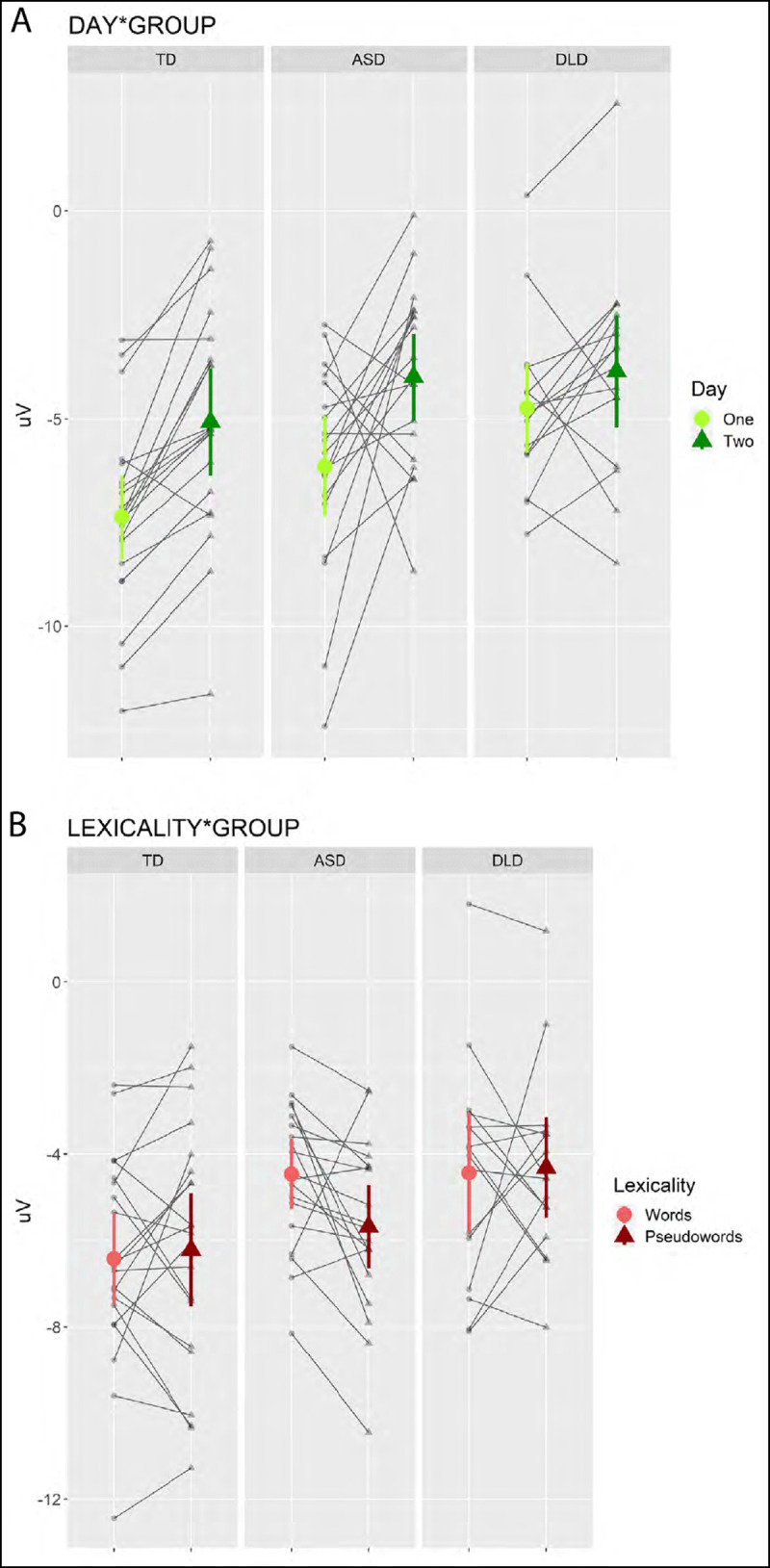
Interactions between **a**) Day and Group **b**) Lexicality and Group, with mean values given; error bars show 95% CI.

Hypothesis 3 stated that *The encoding of novel words within sessions will be less evident in children with DLD compared to typically developing peers, indexed by reduced habituation of the N400 in response to novel words*, such that an interaction was expected between Group, Presentation and Lexicality. This interaction term was removed due to rank deficiency, which indicates insufficient data were available to assess each unique term in the model, and replaced with two simpler interactions: Group by Presentation, to assess whether the hypothesis could be supported across conditions, and Group by Lexicality, to assess whether the hypothesis could be supported across presentations. The Group:Presentation term did not emerge as significant. Group:Lexicality did (see ***Figure 5b***), but with only the ASD group differing in their response to Words (mean = –4.49 µV, *SE* = 0.18) and Pseudowords (mean = –5.64 µV, *SE* = 0.18): *b* = 1.40, *SE* = 0.31, *z* = 4.488, *p* < 0.001. Although we would expected to see a smaller lexicality effect if encoding were reduced in the DLD group, to support this hypothesis we would need to see better evidence for a lexicality effect in the TD group.

Finally, hypothesis 4 stated that *Children with ASD will show reduced consolidation of novel items between sessions, indexed by reduced habituation of the N400 between days compared to the typically developing and DLD groups*, such that an interaction between Group, Day and Lexicality was expected. This three way interaction was removed due to rank deficiency, and again replaced with a simpler two way interaction term, between Group and Day to test a group-specific change in amplitude between sessions irrespective of stimulus type. This interaction did emerge as significant, however, it was driven by the TD and ASD groups showing a greater change between sessions than compared to the DLD group (see ***Figure 5a***), though the Day contrast was significant for all groups (TD: *b* = –2.08, *SE* = 0.25, *z* = –8.310, *p* < 0.001; ASD: *b* = –2.48, *SE* = 0.26, *z* = –9.702, *p* < 0.001; DLD: *b* = –0.94, *SE* = 0.33, *z* = –2.851, *p* = 0.004. Hypothesis 4 was therefore not supported.

#### Random effects

While both Subject and UP contributed to the models, UP explains very little of the error term, with UP-averaged responses varying from the overall mean with a standard deviation of around half that of Subject-averaged responses in each model. This suggests that the effects we see here relate to a broad epoch and are not influenced substantially by the uniqueness point of items.

## Summary

This study assessed changes in the auditory N400 to novel and known words within and between two ERP sessions spaced 24 hours apart, in children with DLD or ASD compared to language-typical peers. The aim was to consider early changes in neural response to novel phonological forms in two groups of children who have difficulties with the acquisition of new vocabulary, seemingly for different reasons.

Although none of our hypotheses were supported, the data suggest that neural responses to novel and known words evolve over a 24-hour period, and that such changes (as well as responses overall) are diminished in children coming to the task with atypical lexical networks.

In negative-going activity at 400–500 ms post stimulus onset we saw a main effect of day, with responses reducing in amplitude and becoming less negative on the second day of testing. Extant evidence suggests that the N400 represents lexical retrieval (Delogu et a., 2019; Lau et al., 2008), with reduction of amplitude reflecting greater ease of retrieving stored conceptual knowledge through the activation of possible lexical items (see Kutas & Federmeier, 2011). Under this view, the current findings are consistent with the idea that sleep supports the establishment of new phonological representations within the mental lexicon (Davis & Gaskell, 2009). Notably, while research on the role of sleep in word learning informed the design of this work, we cannot directly attribute findings to sleep as we did not take direct objective measurements of sleep parameters.

Children with DLD showed a smaller difference in N400 amplitude between days than those with ASD or typical development. This suggests reduced pre-activation on the second day in those with DLD. A main effect of group was also observed, with the DLD group showing a less negative response overall compared to the typically developing group. These group differences may both reflect reduced lexical search in children with language disorder on account of a sparser lexical network and fewer semantic links (Sheng & McGregor, 2010). This interpretation does not align well with the idea that reduced N400 amplitude reflects easier lexical access. However, N400 amplitude is known to be sensitive to multiple linguistic manipulations, being, for example, larger (more negative) in response to words with high semantic richness (Kounios et al., 2009) and words with large phonological neighbourhoods (Dufour et al., 2012). For children with DLD, the stimuli presented here were likely to have fewer phonological neighbours and also be lower in semantic richness compared to that same stimulus set for children with better language skills.

As expected (Helenius et al., 2009; 2014), a main effect of condition emerged, with pseudowords evoking a more negative response than words. However, this lexicality effect was driven by the children with ASD rather than those with typical development, making this finding difficult to interpret. We speculate here that a reduced lexicality effect in the children with typical development may reflect word-like pseudoword stimuli (such as ‘eletrop’) activating existing representations in this group. Nonwords derived from real words are known to activate the semantic representations of their root words (Chuang et al., 2021; Rosson, 1983). We might expect reduced ease of access to the lexical representations of root words in children with ASD, given previous N400 studies that have shown limited integration of heard words with their meaning (DiStefano et al., 2019), a finding which may be specific to the linguistic domain (McCleery et al., 2010).

The absence of clear habituation effects may be a result of our paradigm design. We asked children to respond to catch trials that were unrelated to the experimental stimuli. This was intended to maximise attentional capacity in children and minimise motor preparedness ERP responses. However, as habituation is attenuated or eliminated for unattended stimuli for auditory ERP components later in the waveform (Hsu et al, 2014), our manipulation may have had the inadvertent effect of reducing attention to the experimental stimuli.

### Limitations and future work

Although the general phenomenon of neural habituation is well established, in future replication and extension studies, children should be required to attend to the target stimuli in order to invoke repetition suppression, which can then be used as a marker for change in the representation of new lexical items more clearly. The attention issues we observed in the DLD group were significant, and clearly affected the quality of our data set from that group. In general, the use of the dry electrode EEG system, while it had enormous benefits in terms of flexibility of testing, did result in lower signal-to-noise ratio than would be expected in a laboratory setting (Grummett et al., 2015; Radüntz, 2018), with movement artefacts being a substantial issue.

### Conclusions

This work provides electrophysiological support for the idea that new phonological representations change over time and that such change may be more limited in children with sparser lexical networks. We saw weaker (less negative) responses in children with DLD and reduced change overnight compared to peers with TD or ASD, suggesting that children with DLD showed less pre-activation of lexical representations in response to words encountered the previous day, such that lexical access was facilitated to a smaller degree. We saw a lexicality effect emerge in children with ASD, but not their typically developing peers, which we speculate is due to word-like stimuli activating existing representations in children with typical development.

In order to improve word learning support for children with any developmental disorder of communication it is vital to understand where that process is sub-optimal for different groups of individuals. We therefore believe that work on the relationships between changing brain-level responses to new word forms and sleep architecture in children for whom trajectories of word learning differ will make important contributions to clinical understanding of vocabulary acquisition.

## Data Accessibility Statement

The datasets used and analysed during the current study, along with analysis scripts, are available at [*https://osf.io/jcgv9/files/*].

## Additional File

The additional file for this article can be found as follows:

10.5334/joc.204.s1 Supplementary Materials. Tables SM1 and SM2.

## References

[1] Bates, D., Maechler, M., Bolker, B., & Walker, S. (2015). Fitting linear mixed-effects models using lme4. Journal of Statistical Software, 67(1), 1–48. DOI: 10.18637/jss.v067.i01

[2] Bishop, D. V. M. (2003). The Children’s Communication Checklist, 2nd Edition. London: Pearson.

[3] Bishop, D. V. M., Barry, J. G., & Hardiman, M. J. (2012). Delayed retention of new word-forms is better in children than adults regardless of language ability: A factorial two-way study. PloS one, 7(5), e37326. DOI: 10.1371/journal.pone.0037326 22615979 PMC3353950

[4] Bishop, D. V. M., & Hsu, H. J. (2015). The declarative system in children with specific language impairment: a comparison of meaningful and meaningless auditory-visual paired associate learning. BMC Psychology, 3, 3. DOI: 10.1186/s40359-015-0062-7 25780564 PMC4342083

[5] Bishop, D., Snowling, M., Thompson, P., Greenhalgh, T., & the CATALISE consortium. (2016). CATALISE: a multinational and multidisciplinary delphi consensus study. Identifying language impairments in children. PLOS ONE, 11(12), e0168066. DOI: 10.1371/journal.pone.0168066 27392128 PMC4938414

[6] Chuang, Y. Y., Vollmer, M. L., Shafaei-Bajestan, E., et al. (2021). The processing of pseudoword form and meaning in production and comprehension: A computational modeling approach using linear discriminative learning. Behaviour Research, 53, 945–976. DOI: 10.3758/s13428-020-01356-w PMC8219637 32377973

[7] Davis, M., & Gaskell, M. G. (2009). A complementary systems account of word learning: neurla and behavioural evidence. Philosophical Transactions of Royal Society of London B: Biological Sciences, 364(1536), 3773–3800. DOI: 10.1098/rstb.2009.0111 PMC2846311 19933145

[8] Delogu, F., Brouwer, H., & Crocker, M. W. (2019). Event-related potentials index lexical retrieval (N400) and integration (P600) during language comprehension. Brain & Cognition, 135, 103569. DOI: 10.1016/j.bandc.2019.05.007 31202158

[9] DiStefano, C., Senturk, D., & Jeste, S. S. (2019). ERP evidence of semantic processing in children with ASD. Developmental Cognitive Neuroscience, 36, 100640. DOI: 10.1016/j.dcn.2019.100640 30974225 PMC6763343

[10] Dufour, S., Brunellière, A., & Frauenfelder, U. H. (2012). Tracking the time course of word-frequency effects in auditory word recognition with event related potentials. Cognitive Science, 37(3), 489–507. DOI: 10.1111/cogs.12015 23163763

[11] Dumay, N., & Gaskell, M. G. (2007). Sleep-associated changes in the mental representation of spoken words. Psychological Science, 18(1), 35–39. DOI: 10.1111/j.1467-9280.2007.01845.x 17362375

[12] Dunn, L. M., Dunn, D. M., Styles, B., & Sewell, J. (2009). The British Picture Vocabulary Scale III – 3rd Edition. London: GL Assessment.

[13] Elliott, C. D., & Smith, P. (2011). The British Ability Scales, 3rd Edition. London: GL Assessment.

[14] Fletcher, F., Knowland, V. C. P., Walker, S., Gaskell, M. G., Norbury, C., & Henderson, L.-M. (2020). Atypicalities in sleep and semantic consolidation in autism. Developmental Science, 23(3), e12906. DOI: 10.1111/desc.12906 31569286 PMC7187235

[15] Gagnepain, P., Henson, R. N., & Davis, M. H. (2012). Temporal predictive codes for spoken words in auditory cortex. Current Biology, 22, 615–621. DOI: 10.1016/j.cub.2012.02.015 22425155 PMC3405519

[16] Gathercole, S., & Baddeley, A. (1990). Phonological memory deficits in language disordered children: is there a causal connection? Journal of Memory and Language, 29(3), 336–360. DOI: 10.1016/0749-596X(90)90004-J

[17] Gathercole, S. E., Hitch, G. J., Service, E., & Martin, A. J. (1997). Phonological short-term memory and new word learning in children. Developmental Psychology, 33(6), 966–979. DOI: 10.1037/0012-1649.33.6.966 9383619

[18] Gilliam, J. E. (2013). Gilliam Autism Rating Scale – 3^rd^ Edition. London, UK: Pearson.

[19] Grill-Spector, K., Henson, R., & Martin, A. (2006). Repetition and the brain: neural models of stimulus-specific effects. Trends in Cognitive Science, 10, 14–23. DOI: 10.1016/j.tics.2005.11.006 16321563

[20] Grummett, T. S., Leibbrandt, R. E., Lewis, T. W., DeLosAngeles, D., Powers, D. M. W., Willoughby, J. O., Pope, K. J., & Fitzgibbon, S. P. (2015). Measurement of neural signals from inexpensive, wireless and dry EEG systems. Physiological Measurement, 36, 1469–1484. DOI: 10.1088/0967-3334/36/7/1469 26020164

[21] Helenius, P., Parviainen, T., Paetau, R., & Salmelin, R. (2009). Neural processing of spoken words in specific language impairment and dyslexia. Brain, awp134. DOI: 10.1093/brain/awp134 19498087

[22] Helenius, P., Sionen, P., Parviainen, T., Isoaho, P., Hannus, S., Kauppila, T., Salmelin, R., & Isotalo, L. (2014). Abnormal functioning of the left temporal lobe in language-impaired children. Brain & Language, 130, 11–18. DOI: 10.1016/j.bandl.2014.01.005 24568877

[23] Henderson, L. M., Powell, A., Gaskell, M. G., & Norbury, C. (2014). Consolidation of vocabulary in autism spectrum disorder. Developmental Science. Early on-line view, 2014 Mar 17. DOI: 10.1111/desc.12169 24636285

[24] Henderson, L. M., Weighall, A. R., Brown, H., & Gaskell, G. (2012). Consolidation of vocabulary is associated with sleep in children. Developmental Science, 15(5), 674–687. DOI: 10.1111/desc.12639 22925515

[25] Holcomb, P. J., Coffey, S. A., & Neville, H. J. (1992). Visual and auditory sentence processing: a developmental analysis using event-related potentials. Developmental Neuropsychology, 8(2&3), 203–241. DOI: 10.1080/87565649209540525

[26] Holcomb, P. J., & Neville, H. J. (1990). Auditory and visual semantic priming in lexical decision: a comparison using event-related potentials. Language and Cognitive Processes, 5(4), 281–312. DOI: 10.1080/01690969008407065

[27] Hsu, Y.-F., Hämäläinen, J. A., & Waszak, F. (2014). Repetition suppression comprises both attention-independent and attention-dependent processes. NeuroImage, 98, 168–175. DOI: 10.1016/j.neuroimage.2014.04.084 24821530

[28] Junge, C., Boumeester, M., Mills, D. L., Paul, M., & Cosper, S. H. (2021). Development of the N400 for word learning in the first 2 years of life: a systematic review. Frontiers in Psychology. DOI: 10.3389/fpsyg.2021.689534 PMC8277998 34276518

[29] Junge, C., Cutler, A., & Hagoort, P. (2012). Electrophysiological evidence of early word learning. Neuropsychologia, 50(14), 3702–3712. DOI: 10.1016/j.neuropsychologia.2012.10.012 23108241

[30] Kounios, J., Green, D. L., Payne, L., Fleck, J. I., Grondin, R., & McRae, K. (2009). Semantic richness and the activation of concepts in semantic memory: evidence from event related potentials. Brain Research, 1282, 95–102. DOI: 10.1016/j.brainres.2009.05.092 19505451 PMC2709703

[31] Kuperman, V., Stadthagen-Gonzalez, H., & Brysbaert, M. (2012). Age-of-acquisition ratings for 30,000 English words. Behavior Research Methods, 44(4), 978–990. DOI: 10.3758/s13428-012-0210-4 22581493

[32] Kornilov, S. A., Magnuson, J. S., Rakhlin, N., Landi, N., & Grigorenko, E. L. (2015). Lexical processing deficits in children with developmental language disorder: an event-related potential study. Development and Psychopathology, 27, special issue 2: Neural Plasticity, Sensitive Periods and Psychopathology, 459–476. DOI: 10.1017/S0954579415000097 PMC4606961 25997765

[33] Kutas, M., & Federmeier, K. D. (2011). Thirty years and counting: Finding meaning in the N400 component of the event related brain potential (ERP). Annual Review of Psychology, 62, 621–647. DOI: 10.1146/annurev.psych.093008.131123 PMC4052444 20809790

[34] Landi, N., Malins, J. G., Frost, S. J., Magnuson, J. S., Molfese, P., Ryherd, K., Rueckl, J. G., Mencl, W. E., Pugh, K. R. (2018). Neural representations for newly learned words are modulated by overnight consolidation, reading skill, and age. Neuropsychologia, 111, 133–144. DOI: 10.1016/j.neuropsychologia.2018.01.011 29366948 PMC5866766

[35] Lau, E., Phillips, C., & Poeppel, D. (2008). A cortical network for semantics: (de)constructing the N400. Nature Reviews Neuroscience, 9, 920–933. DOI: 10.1038/nrn2532 19020511

[36] Length, R. (2019). emmeans: Estimated Marginal Means, aka Least-Squares Means. R package version 1.3.5. https://CRAN.R-project.org/package=emmeans

[37] Marini, A., Ozbič, M., Magni, R., & Valeri, G. (2020). Toward a definition of the linguistic profile of children with Autism Spectrum Disorder. Frontiers in Psychology, 11, 808. eCollection 2020. DOI: 10.3389/fpsyg.2020.00808 32431644 PMC7214763

[38] McCleery, J. P., Ceponiene, R., Burner, K. M., Townsend, J., Kinnear, M., & Schreibman, L. (2010). Neural correlates of verbal and nonverbal semantic integration in children with autism spectrum disorders. The Journal of Child Psychology and Psychiatry, 51(3), 277–286. DOI: 10.1111/j.1469-7610.2009.02157.x 20025622

[39] McLaughlin, J., Osterhout, L., & Kim, A. (2004). Neural correlates of second-language word learning: minimal instruction produces rapid change. Nature Neuroscience, 7, 703–704. DOI: 10.1038/nn1264 15195094

[40] Minamoto, H., Tachibana, H., Sugita, M., & Okita, T. (2001). Recognition memory in normal aging and Parkinson’s disease: behavioral and electrophysiologic measures. Cognitive Brain Research, 11(1), 23–32. DOI: 10.1016/S0926-6410(00)00060-4 11240108

[41] Norbury, C. F., Griffiths, H., & Nation, K. (2010). Sound before meaning: word learning in autistic disorders. Neuropsychologia, 48(14), 4012–4019. DOI: 10.1016/j.neuropsychologia.2010.10.015 20951710

[42] Nordt, M., Hoehl, S., & Weigelt, S. (2016). The use of repetition suppression paradigms in developmental cognitive neuroscience. Cortex, 80, 61–75. DOI: 10.1016/j.cortex.2016.04.002 27161033

[43] Owens, J. A., Spirito, A., & McGuinn, M. (2000). The Children’s Sleep Habits Questionnaire (CSHQ): psychometric properties of a survey instrument for school-aged children. Sleep, 23(8), 1043–1051. DOI: 10.1093/sleep/23.8.1d 11145319

[44] R Core Team. (2017). R: A language and environment for statistical computing. Vienna, Austria: R Foundation for Statistical Computing. URL https://www.R-project.org/

[45] Radüntz, T. (2018). Signal quality evaluation of emerging EEG devices. Frontiers in Physiology, 14>. DOI: 10.3389/fphys.2018.00098 PMC5817086 29491841

[46] Roediger, H. L., III & Butler, A. C. (2011). The critical role of retrieval practice in long term retention. Trends in Cognitive Sciences, 15, 20–27. DOI: 10.1016/j.tics.2010.09.003 20951630

[47] Rosson, M. B. (1983). From SOFA to LOUCH: Lexical contributions to pseudoword pronunciation. Memory and Cognition, 11, 152–160. DOI: 10.3758/BF03213470 6865749

[48] Rugg, M. (1985). The effects of semantic priming and word repetition on event-related potentials. Psychophysiology, 22(6), 642–747. DOI: 10.1111/j.1469-8986.1985.tb01661.x 4089090

[49] Rugg, M. (1990). Event-related brain potentials dissociate repetition effects of high and low frequency words. Memory and Cognition, 18(4), 367–379. DOI: 10.3758/BF03197126 2381316

[50] Sheng, L., & McGregor, K. (2010). Lexical-semantic organisation in children with specific language impairment. Journal of Speech, Language and Hearing Research, 53(1), 146–159. DOI: 10.1044/1092-4388(2009/08-0160) PMC3328209 20150406

[51] The MathWorks. (2014). MATLAB Release 2014. Natick, Massachusetts, United States: The MathWorks, Inc.

[52] Volpert-Esmond, H. I., Merkle, E. C., Levsen, M. P., Ito, T. A., & Bartholow, B. D. (2018). Using trial-level data and multilevel modelling to investigate within-task change in event-related potentials. Psychophysiology, 55(5), e13044. DOI: 10.1111/psyp.13044 29226966 PMC5899682

[53] Wagner, R., Torgesen, J., Rashotte, C. A., & Pearson, N. A. (2013). Comprehensive test of phonological processing, 2nd Edition (CTOPP2). Oxford: Pearson. DOI: 10.1037/t52630-000

[54] Wickham, H. (2016). ggplot2: Elegant Graphics for Data Analysis. New York: Springer-Verlag. DOI: 10.1007/978-3-319-24277-4

[55] Williams, D., Botting, N., & Boucher, J. (2008). Language in autism and specific language impairment: where are the links? Psychological Bulletin, 134(6), 944–963. DOI: 10.1037/a0013743 18954162

[56] Williams, D., Payne, H., & Marshall, C. (2012). Non-word repetition impairment in autism and specific language impairment: evidence for distinct underlying cognitive causes. Journal of Autism and Developmental Disorders, 43(2), 404–417. DOI: 10.1007/s10803-012-1579-8 22733298

