# Supplementary material for: Neural Responses to Novel and Existing Words in Children with Autism Spectrum and Developmental Language Disorder
Source: J Cogn. 2022 Jan 27;5(1):14. doi: 10.5334/joc.204 (PMC9400667; doi:10.5334/joc.204)
Supplement: Supplementary Materials. — Tables SM1 and SM2. [file joc-5-1-204-s1.pdf]

SUPPLEMENTARY MATERIALS

**Words**		**Pseudo-words**	
**Orthography**	**Phon.DISC**	**Orthography**	**Phon.DISC**
aeroplane	'**8r@**pl1n	aerobrise	'**8r@**br2z
alligator	'**{lI**g1t@R	alibodo	'**{lI**b5d5
ambulance	'**{mb**jUl@ns	ambairston	'**{mb**8rstQn
apricot	'**1pr**IkQt	aprodin	'**1pr**@dIn
astronaut	'**{st**r@n$t	astorbol	'**{st**$rb5l
badminton	'**b{d**mInt@n	badresmit	'**b{d**r@smIt
balcony	'**b{l**k@nI	balzipor	'**b{l**zIp$r
ballerina	**b{l@**'rin@	ballenodo	**b{l@**'n5d5
banana	**b@**'n#n@	barazo	**b@**'r{z5
binoculars	**bI'n**QkjUl@z	binitozart	**bI'n**it5z#t
broccoli	'**brQ**k@lI	brotifa	'**brQ**tIf@
buffalo	'**bVf**@l5	buffishie	'**bVf**ISi
calendar	'**k{l**Ind@R	kalipros	'**k{l**ipr5s
caravan	'**k{r@**v{n	karalom	'**k{r@**l5m
casserole	'**k{s**@r5l	kasiveen	'**k{s**Ivin
caterpillar	'**k{t@p**Il@R	caterpozie	'**k{t@p**Qzi
cauliflower	'**kQlI**fl6@R	caulibeon	'**kQlI**bI5n
computer	**k@m'p**jut@R	compingle	**k@m'p**INgP
crocodile	'**krQ**k@d2l	crozigeri	'**krQ**zIg@i
cucumber	'**kju**kVmb@R	cubingi	'**kju**biN_i
daffodil	'**d{f**@dIl	daforon	'**d{f**$rQn
dandelion	'**d{ndI**l2@n	dandisomo	'**d{ndI**s5m5
dinosaur	'**d2n**5sOR	dinepi	'**d2n**Ipi
elephant	'**ElI**f@nt	eletrop	'**ElI**tr5p
flamingo	**fl@**'mINg5	flasompie	**fl@**'s5mpi
gorilla	**g@**'rIl@	gewano	**g@**'w{n5
helicopter	'**hElI**kQpt@R	helneseepo	'**hEl**n@sip5
hurricane	'**hVr**Ik1n	hurorbive	'**hVr**$rbiv
kangaroo	**k{**Ng@'ru	kapmisi	**k{**pmI'si
macaroni	**m{k**@'r5nI	makorteemo	**m{k**$'tIm5
marshmallow	**m#S**'m{l5	marshpiena	**m#S**'p2n@
mayonnaise	**m1**@'n1z	mayvetor	**m1**v@'t$r
mosquito	**m@s**'kit5	mosgumi	**m@s**'gumi
pelican	'**pEl**Ik@n	pelonog	'**pEl**@n5g
potato	**p@**'t1t5	pehoili	**p@**'h
pyjamas	**p@**'_#m@z	perigin	**p@**'rigIn
pyramid	'**pIr**@mId	pyrobam	'**pIr**5b{m
raspberry	'**r#z**b@rI	rarzwipa	'**r#z**wip@
rectangle	'**rEkt**{NgP	rectossen	'**rEkt**5sEn
scorpion	'**sk$**pj@n	skorlaior	'**sk$**l1$r
skeleton	'**skE**lItH	skepinel	'**skE**pInL
spaghetti	**sp@**'gEtI	spaligo	**sp@**'lig5
tambourine	**t{**mb@'rin	tagriboof	**t{**gri'buf
tarantula	**t@'r**{ntjUl@	tariksonot	**t@'r**Iks@n5t
television	**tElI**'vIZH	telijuvo	**tElI**'_uv5
umbrella	**Vm**'brEl@	umponot	**Vm**'p5n$t
vanilla	**v@**'nIl@	vapoko	**v@**'p5k5
vegetable	'**vE_**t@bP	vegeroon	'**vE_**Erun
vinegar	'**vI**nIg@R	vizigad	'**vI**zig{d
vitamin	'**vI**t@mIn	vipient	'**vI**pi@nt

Table SM1. Stimuli. After the uniqueness point no Pseudoword had a phoneme in the same place as in its counterpart Word, though the two were matched for syllable and phoneme number, stress, and phonotactic probability. For each Word, uniqueness point was determined using the Celex database taking intonation and age-of-acquisition into account. All Word stimuli had at least two phonemes before the uniqueness point and at least two after.

	Syll.	Phon.	UP	Phon. After UP	Freq.	AoA	Concrete	Phon. prob
Mean	3.2	7.6	4.0	3.7	10.5	6.1	4.9	1.3
Min.	3	6	3	2	1	3.8	4.4	1.2
Max.	4	10	6	6	114	10.9	5.0	1.4

Table SM2. Characteristics of Word stimuli: the number of *Syllables* and *Phonemes;* the number of phonemes before the uniqueness point (*UP*), the number of *Phonemes After UP; Frequency* of occurrence per million using the SUBTLEX-US frequency norms of Brysbaert & New, 2009; Age of acquisition (*AoA*) from Kuperman et al., 2012; *Concreteness* ratings from Brysbaert, Warriner & Kuperman, (2014); and mean Phonotactic Probability for the items, using an online calculator by Vitevitch, and Luce (2004).
